# Feasibility Study of Enhancing Microwave Brain Imaging Using Metamaterials

**DOI:** 10.3390/s19245472

**Published:** 2019-12-12

**Authors:** Eleonora Razzicchia, Ioannis Sotiriou, Helena Cano-Garcia, Efthymios Kallos, George Palikaras, Panagiotis Kosmas

**Affiliations:** 1 Faculty of Natural and Mathematical Sciences, King’s College London, Strand, London WC2R 2LS, UK; ioannis.sotiriou@kcl.ac.uk; 2 MediWise Ltd., London E1 2AX, UK; helena.cano_garcia@kcl.ac.uk (H.C.-G.); themos.kallos@mediwise.co.uk (E.K.); george.palikaras@mediwise.co.uk (G.P.)

**Keywords:** metamaterial, metasurface, split-ring resonators (SRRs), cross-shaped split-ring resonators (CS-SRRs), microwave tomography (MWT), microwave imaging (MWI), brain imaging

## Abstract

We present an approach to enhance microwave brain imaging with an innovative metamaterial (MM) planar design based on a cross-shaped split-ring resonator (SRR-CS). The proposed metasurface is incorporated in different setups, and its interaction with EM waves is studied both experimentally and by using CST Microwave Studio^®^ and is compared to a “no MM” case scenario. We show that the MM can enhance the penetration of the transmitted signals into the human head when placed in contact with skin tissue, acting as an impedance-matching layer. In addition, we show that the MM can improve the transceivers’ ability to detect useful “weak” signals when incorporated in a headband scanner for brain imaging by increasing the signal difference from a blood-like dielectric target introduced into the brain volume. Our results suggest that the proposed MM film can be a powerful hardware advance towards the development of scanners for brain haemorrhage detection and monitoring.

## 1. Introduction

Brain-related diseases and disorders are an enormous burden for healthcare systems and people [1]. The need for minimally invasive and personalised technologies for the brain is a challenge involving several research fields. In this context, new electromagnetic (EM) technologies for brain disease diagnostics and monitoring are receiving increased attention [2]. For example, magnetoencephalography (MEG) is a functional neuroimaging technique for mapping brain activity, in which magneto-impedance (MI) sensors are used to measure the head’s biomagnetic field [3].

Among these emerging EM techniques, microwave tomography (MWT) has great diagnostic potential in various clinical applications such as breast cancer detection, stroke detection, cardiac imaging, bone imaging, and localization of in-body radio frequency (RF) sources [4,5,6]. This imaging technique is based on differentiation of dielectric properties of the biological tissue under study [7]. Array of antennas are used to transmit low-power microwaves in the 0.5–3.0 GHz frequency range into the region of interest and to receive the resulting scattered signal. The spatial distribution of dielectric properties throughout the tissue volume is then estimated using reconstruction algorithms that are applied to the acquired data [8].

Although microwave imaging (MWI) methods initially focused on diagnosing breast cancer, MWI techniques for brain stroke detection and classification are drawing the interest of many groups worldwide [9,10,11,12,13]. Another possible application of MWI techniques is in detecting traumatic intracranial bleedings [14]. Currently, intracranial hematoma identification and stroke detection and classification rely on imaging methods such as computed tomography (CT) and magnetic resonance imaging (MRI). For example, CT is able to confirm the diagnosis and to tell whether a stroke is ischemic (i-stroke), e.g., a blood clot restricts the blood flow, or haemorrhagic (h-stroke), e.g., a blood vessel bursts and bleeds in the brain, whereas MRI can identify and further localize the stroke’s site. However, these two technologies are characterized by high costs and low accessibility. In addition, both these tools are not portable and thus cannot be easily used by paramedics or be found in ordinary ambulances [15]. In contrast, MWI uses non-ionizing radiation, is a noninvasive technique, and can lead to low-cost and portable diagnostic devices. Moreover, its data acquisition times can range from milliseconds to a few seconds, which can allow a quick assessment of the stroke, which is crucial for proper drug administration [12].

Besides all the advantages of MWT, developing a valid apparatus for brain imaging is a challenging task. For example, to achieve an operational microwave imaging (MWI) system for detecting a bleeding in the brain that could be due to a traumatic injury or the rupture of a blood vessel in the brain (h-stroke), it is necessary to find a compromise between resolution and penetration depth into the brain tissue, which is located inside of a shield comprising the skull and the cerebrospinal fluid [16]. Thus, a valid MWI system should maximize the incident power coupled into the tissue of interest and should ensure acceptable spatial resolution images [17]. To maximize the amount of incident power penetrating into the brain tissue, compact antennas operating below 2.0 GHz immersed in a lossy dielectric medium are recommended due to the strong attenuation of the EM waves propagating inside the head [17]. According to this requirement, there are several possible MWI head scanner designs that could be considered. Headband, helmet structures, or specific chambers [18], for example, cover only portion of the head to allow the use of a coupling medium for the operating antennas. The dielectric medium reduces unwanted multipath signals [19] and broadens the frequency range of operation [20] but, at the same time, affects the detection of useful “weak” signals due to the target. One way to increase the transmitted power and to improve the received signal is to place a metamaterial (MM) film in front of the skin, which can act as an antireflection coating [21,22]. This approach is motivated by previous numerical studies which have demonstrated that planar MMs, also called metasurfaces, can suppress unwanted reflections and can enhance the transmission near a specific frequency [23,24].

An MM is an engineered electromagnetic structure with particular properties, such as negative permittivity and permeability (that translates into a negative refractive index), which are not found in naturally occurring materials. Pendry et al. have first demonstrated that microstructures built from nonmagnetic conducting sheets exhibit an effective magnetic permeability not accessible in natural materials [25]. Furthermore, they have shown that most of these microstructures are resonant due to internal capacitance and inductance [26]. These metamaterial structures are based on sub-wavelength resonators, mainly split ring resonators (SRRs). Nowadays, different SRR structures have been developed for several microwave applications including sensing and imaging, as they present many advantages relative to conventional structures such as high flexibility in the design process [27]. MMs can also lead to innovative structures for radiating and scattering applications by taking advantage of their ability to manipulate the size, efficiency, bandwidth, and directivity of different systems [28].

In the microwave spectrum, layers of SRR have been used for biosensing for cancer detection [29,30], magnetic resonance imaging [31,32], and imaging in the near-field [33,34] and far-field [35]. Compact planar MMs based on SRRs have been also used to enhance the performance of antennas for MWI applications [36,37]. At higher frequencies, MM substrates have been proposed to improve the antenna gain and directivity for in-vivo THz biomedical applications [38,39]. Imaging systems for security-screening applications have also used frequency-diverse metasurface apertures in the K-band frequencies [40,41,42].

Motivated by an important potential medical application, this paper investigates the feasibility to improve the accuracy of brain haemorrhage detection and localization with the use of an innovative MM technology. To this end, this paper presents experiments with simplified planar phantoms and a cylindrical target mimicking bleeding in the brain as well as numerical simulations with a tomographic scanner and a brain model with a target. Our SRR-based MM film is first proposed as an antireflection coating able to improve impedance matching and, thus, to reduce reflection and to enhance transmission of microwave energy into a body region such as the head [43]. However, aware that a slight increase in overall transmission may not necessarily improve an MWI system’s ability to detect bleeding in the brain, this paper focuses on examining whether the MM can enhance the “weak” signal scattered from a blood-like target, which is relevant to our application of interest. To this end, we present results from simulations and experiments which suggest that the MM film can have a positive impact when placed on the head as an impedance matching layer or closely fixed to the MWI array’s antennas, leading to higher signals scattered from the target. Moreover, we present reconstructions from data produced by simulations of a microwave scanner probing a numerical head model with a blood-like target, which show that more accurate detection and localization of the target is achieved in the presence of the MM.

The remainder of the paper is organized as follows: Section 2 presents the methodology for the unit cell design and two proposed MM layers. It also describes the geometry of a two-port experimental setup including the MM and shows how it might be integrated in our headband scanner for brain imaging. Section 3 presents signals from simulations and experiments, which suggest that the use of the MM not only enhances signal transmission but also, more importantly increases the MWI system’s sensitivity to the signal scattered by the target that we wish to detect. To illustrate this further, we compare MWT reconstructions using our previously developed DBIM-TwIST algorithm with and without the MM. These images confirm that the MM enhances detection of a blood-like target of interest. Finally, Section 4 includes some discussions, concluding remarks, and future steps.

## 2. Materials and Methods

To validate the EM antireflection coating concept, we have performed several simulation studies to optimize an MM geometry for operating in contact with the human skin when immersed in a 90% glycerol–water mixture. This dielectric medium is required for our system’s antennas to operate efficiently in the desired frequency range [44]. One of our previous studies has already presented the MM unit cell design and the periodic structure based on this pattern [43], but we review and expand this methodology further.

### 2.1. MM Design and Simulations for Impedance Matching

**Figure 1:**
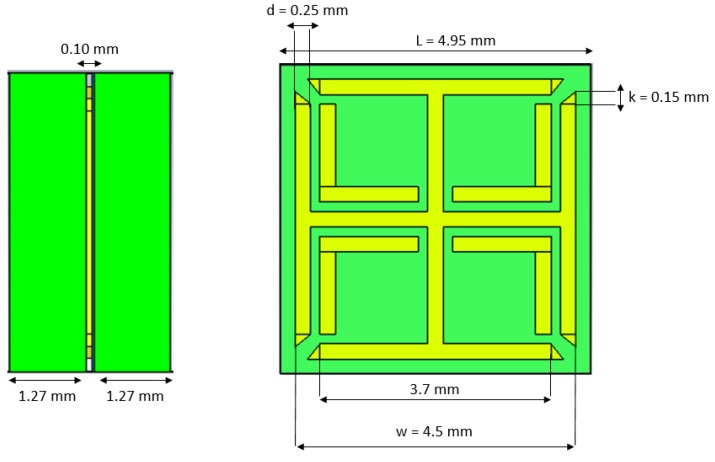
Geometry and dimensions of the proposed metamaterial (MM) unit cell, comprising a metallic pattern sandwiched in between two high dielectric substrates.

We propose an innovative MM design based on a square unit cell, which comprises a copper lattice (thickness = 0.10 mm) embedded between two Rogers high-dielectric substrates that are bonded with Rogers 3001 bonding film (ϵ’ = 2.28, tanδ = 0.003). The metallic pattern design is a variation of the Jerusalem Cross SRR. Its frontal and lateral view are illustrated in Figure 1.

The unit cell was first incorporated in a simplified planar model of the head comprising seven flat tissue layers placed in the following order: skin (thickness $t_{1}$ = 2.8 mm) [45], cortical bone (outer layer) ($t_{2}$ = 1.8 mm), cancellous bone ($t_{3}$ = 2.3 mm), cortical bone (inner layer) ($t_{4}$ = 1.4 mm) [46,47], cerebrospinal fluid (CSF) ($t_{5}$ = 4.2 mm), grey matter ($t_{6}$ = 2.5 mm), and white matter ($t_{7}$ = 56.6 mm) [48]. The dielectric properties of the tissues at 1.0 GHz were taken from Reference [49]. The interaction of this setup with a normally incident plane wave in the 0.5–1.5 GHz frequency range was modelled in CST Microwave Studio^®^. The S-parameters were calculated considering “Port 1” before the glycerol layer (thickness $t_{g}$ = 9 mm, permittivity ϵ’ = 18 and conductivity σ= 1.3 S/m at 1.0 GHz) and “Port 2” after the white matter layer.

**Figure 2:**
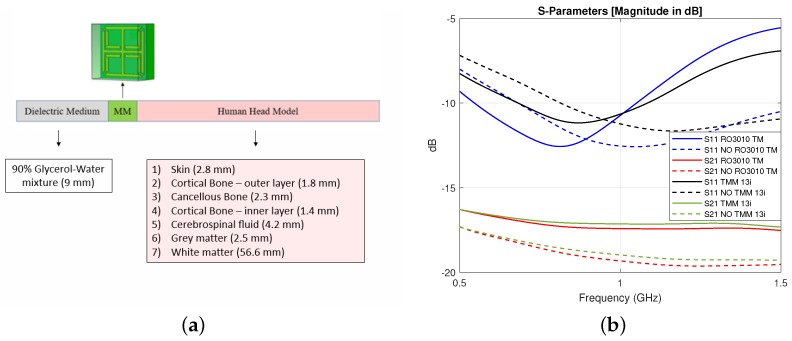
(**a**) Unit cell geometrical configuration and simulation setup: The “human head model” comprises seven tissue layers. (**b**) Simulation results for the transmission $S_{21}$ and reflection $S_{11}$ parameters with (solid lines) and without (dashed lines) the MM for two different substrates: Both Rogers RO3010 TM and Rogers TMM13i led to an improvement in transmission of about 1.80 dB at 1.0 GHz with respect to the “no MM” case.

To optimize the MM unit cell design, the improvement in transmission obtained with different geometries and substrates was studied. In particular, variations in the classic Jerusalem Cross design were introduced in order to achieve a better $S_{21}$ parameter and two substrates were tested: Rogers RO3010 TM (thickness = 1.27 mm, ϵ’ = 10.2 and tanδ = 0.0022) and Rogers TMM 13i (thickness = 1.52 mm, ϵ’ = 12.2 and tanδ = 0.0019). As shown in Figure 2, both the substrates led to an improvement in transmission of about 1.80 dB at 1.0 GHz.

**Figure 3:**
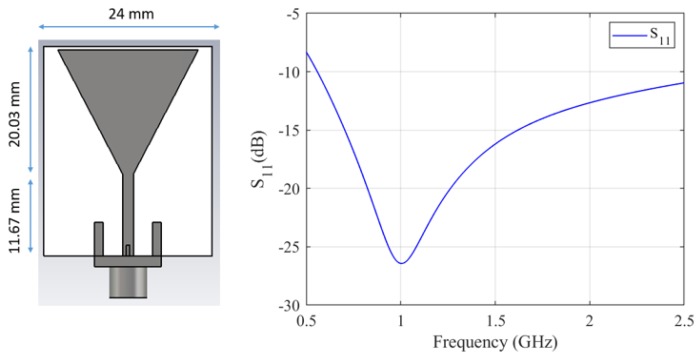
Design and reflection coefficient ($S_{11}$) of the antenna used as excitation source: The antenna consists of a triangular patch on a FR-4 substrate, with a partial ground on the back side. The $S_{11}$ presents a deep resonance at 1.0 GHz, where $S_{11}$ falls below −25 dB. @2019 IEEE [50].

**Figure 4:**
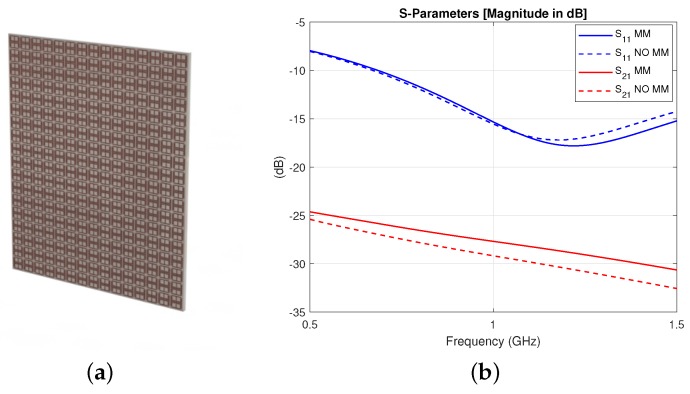
(**a**) Finite MM periodic structure comprising an inner metallic pattern in between two high dielectric substrates and (**b**) simulation results for the transmission $S_{21}$ and reflection $S_{11}$ parameters with (solid lines) and without (dashed lines) the MM, showing an improvement in transmission of about 1.50 dB at 1 GHz in the presence of the finite MM.

Based on the unit-cell results, a periodic structure based on the optimized unit cell geometry was studied, with Rogers 3010 TM as its substrate because of its flexibility. In particular, with the aim of simulating a setup that we could also easily fabricate, a 15 × 18 unit cell MM structure was simulated in contact with a skin slab (thickness $t_{1}$ = 1 mm). The simulation setup included two other tissue layers: cortical bone ($t_{2}$ = 2 mm) and brain ($t_{3}$ = 7 mm). A layer of 90% glycerol–water mixture was added before the MM film, and the antenna proposed in Reference [44] and shown in Figure 3 was used to excite the full setup in the 0.5–1.5 GHz frequency range. The S-parameters for reflection and transmission were calculated for an antenna placed at “Port 1” and “Port 2” before and after the brain model, respectively, and are plotted in Figure 4. The results show that the improvement in transmission caused by the finite MM structure is close to the value calculated with the unit cell approach in the previous section, suggesting that a 15 × 18 unit cell structure can approximate the “infinite” MM sheet for the purposes of our application.

### 2.2. Experimental Validation

**Figure 5:**
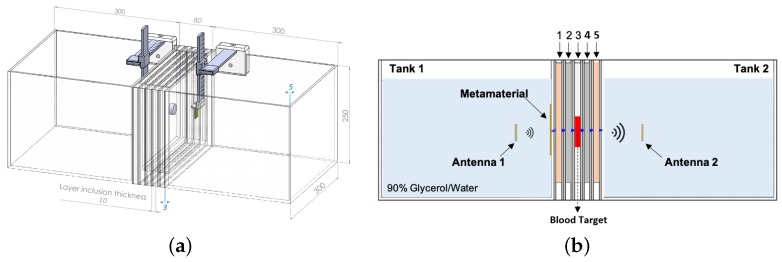
(**a**) Technical drawing showing the geometric dimensions of the setup expressed in millimetres. (**b**) Schematic design showing the setup in the “with target” configuration.

**Figure 6:**
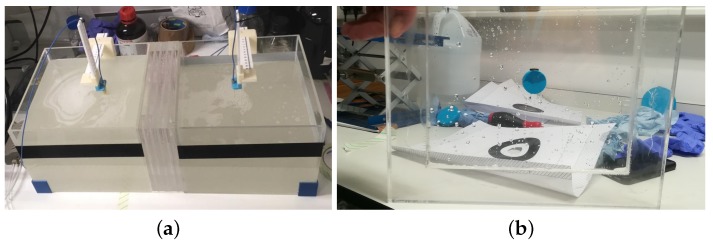
(**a**) Experimental setup and (**b**) target capsule containing horse blood in EDTA (Ethylenediaminetetraacetic Acid) centered in Layer 3.

To study the performance of our MM, the 2-port custom-made experimental setup shown in Figure 5a and Figure 6a was designed and fabricated. This comprises five rectangular acrylic layers (ϵ’ = 2.53, tanδ = 0.0119) of 240-mm width, 215-mm height, and 16-mm thickness which are placed in series in between two acrylic tanks (300 mm × 300 mm × 250 mm). Two antennas, each immersed in a tank, are used to transmit and receive signals in the range of 0.5–4.0 GHz using Keysight’s PXI vector network analyser (M9370A). The tanks are filled with a lossy dielectric medium made of a 90% glycerol–water mixture, which is used as immersion liquid for the employed antennas [44]. The measurements were taken at three equal antenna distances of 100 mm, 110 mm, and 130 mm, whereas the MM was positioned manually on the acrylic surface close to Antenna 1 and guided to the correct position using a custom designed slider to avoid any errors caused by movement. When the antennas are placed at a distance of 100 mm, Antenna 1 is in its closest position to the MM (4 mm from it).

The middle five acrylic layers were filled with various liquid phantoms, which were made to resemble different types of human tissue. These liquid phantoms were easy to fabricate and were suitable for reproducing a simple planar model of the human head. Supported by the acrylic layers, the phantoms were stacked in the following order: skin (“Layer 1”), bone (“Layer 2”), brain or blood (“Layer 3”), bone (“Layer 4”), and skin (“Layer 5”). Each liquid phantom was measured using a dielectric probe connected to the VNA (Vector Network Analyzer). The real part of the measured permittivity at 1 GHz is shown in Table 1.

To investigate the impact of our MM matching layer, we conducted measurements in different experimental scenarios. First, to assess the system’s sensitivity to changes in permittivity of the inner layer (“Layer 3”), we measured transmitted signals without the MM for “Layer 3” filled with different liquids: brain phantom, blood phantom, and horse blood in EDTA (Ethylenediaminetetraacetic Acid) from TCS Biosciences (Botolph Claydon, Buckingham, UK). Subsequently, the same measurements were taken in the presence of the MM and differences in the transmission coefficient $S_{21}$ with and without the MM were calculated and compared. Furthermore, to demonstrate that the MM is able to enhance the signal due to a blood-like target, a cylindrical capsule (25-mm diameter and 8-mm thickness) made of ABS (Acrylonitrile Butadiene Styrene). (ϵ’ = 2.74, tanδ = 0.0051) and filled with horse blood in EDTA was included and centred in the inner layer of the setup. The signal difference “with target – no target” (dB) was measured with and without the MM. We refer to the basic setup configuration (“Layer 3” filled with brain phantom only) as the “no target” scenario. Figure 5b illustrates the “with target” configuration, consisting of “Layer 3” filled with the brain phantom and including the blood capsule.

Representative configurations of the system described above were modelled using CST Microwave Studio^®^ in the 0.5–2.0 GHz frequency range. As the liquid phantoms were correctly resembling the dielectric properties of all the tissues, we assigned the values of permittivity and conductivity at 1.0 GHz from Reference [49]. These values were already acceptable approximations of our phantom’s dielectric properties for our frequency of interest. We used dielectric values based on measurements for the horse blood properties (ϵ’ = 58.7 at 1 GHz). In addition, to further demonstrate the MM’s ability to enhance transmission, a “Negative Control” (NC) case was also considered, for which the MM was replaced by a material with the same dielectric substrate and dimensions but without the metallic pattern. This allowed evaluating the effect due to the metallic pattern design.

### 2.3. Simulations with a Brain Imaging Scanner

**Figure 7:**
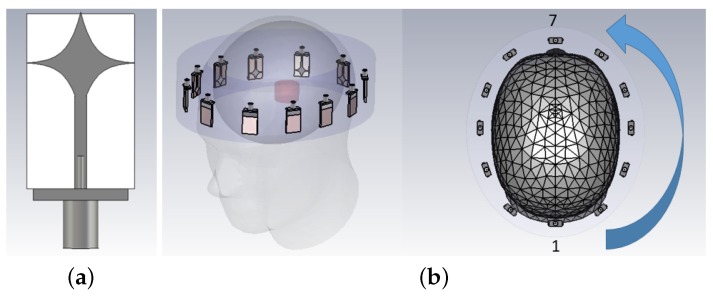
(**a**) Spear-shaped antenna used in the headband system, operating at 1.5 GHz when immersed in the 90% glycerol coupling medium: It consists of a spear patch printed on a FR-4 substrate, with a rectangular ground on the back side. (**b**) Headband system geometry and head model: The array is close to the head, around 10.0 mm on average. The headband is 65.0 mm thick, whereas the major and minor axes of the elliptical cross section are 260 mm and 220 mm, respectively [9]. A MM layer (5 × 6 unit cells) is placed in front of each antenna at different distances to evaluate its effect on the array’s signals and the resulting reconstructed images.

Having examined the MM performance with the two-port planar setup, further simulation studies were performed in CST Microwave Studio^®^ to confirm that integrating the MM in an MWI system would also enhance performance. To this end, the MM was incorporated in an MWT brain scanner proposed for microwave head imaging, consisting of 12 antennas arranged uniformly inside an elliptical headband filled with the 90% glycerol–water matching medium [9]. The scanner was placed around a numerical head phantom, for which the EN 50361 Specific Anthropomorphic Mannequin (SAM) head model was used. This simplified head model includes only a skull layer (bone) and average brain tissue inside. The setup is shown in Figure 7.

We refer to the model with brain and bone alone as the “no target” scenario. We have also studied the signal propagation in the presence of a blood-like target inside the brain volume. The target was a cylinder of 30-mm diameter and 20-mm height and was placed in the vertical centre of the brain in the front-left part close to antennas 8–10 (see Figure 7). We refer to this case (with the blood target introduced in the brain) as the “with target” scenario. In order to generate the reconstruction images with our previous developed DBIM-TwIST algorithm, we used this system geometry, assigning a permittivity value of 20 and conductivity of 0.35 S/m to the bone region and respectively 45.8 and 0.76 S/m to the brain tissue.

A smaller MM layer (5 × 6 unit cells) was placed in front of each antenna at different distances. The MM layer was reduced in size in order to be placed at various distances between the head skull and the array. In particular, we present two case studies: the MM was placed on the head and adjacent to the antenna’s substrate. The S-parameters were measured over the 0.5–2.0 GHz frequency range. Then, the signal difference “with target–no target” (dB) with and without the MM was calculated.

## 3. Results

### 3.1. Experimental and Simulation Results for the Two-Port Planar Setup

**Figure 8:**
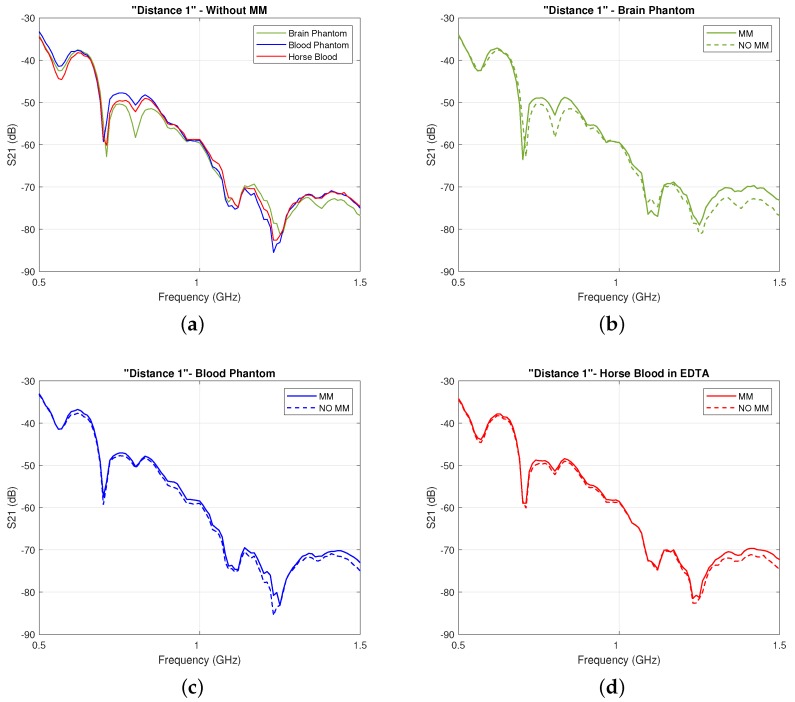
(**a**) Comparison of the experimentally measured transmission coefficient $S_{21}$ for “Distance 1” without the MM and with inner layer filled with brain phantom, blood phantom, and horse blood; (**b**) Comparison of the experimentally measured transmission coefficient $S_{21}$ for “Distance 1” with the MM (solid lines) and without the MM (dashed lines) when the inner layer is filled with the brain phantom, (**c**) with the blood phantom, and (**d**) with the horse blood in EDTA.

First, we present experimental results in Figure 8, which show that the two-port planar system of Figure 6 is sensitive to changes in the dielectric properties of the inner acrylic layer (Figure 8a) and that the MM enhances transmission over the 0.5–1.5 GHz frequency range for all three cases of the inner layer filled with the brain phantom (Figure 8b), blood phantom (Figure 8c), or real horse blood (Figure 8d). We observed that the MM increases the $S_{21}$ coefficient around 1–2 dB over the considered frequency range, with higher differences near 1.5 GHz. Results shown in Figure 8 were obtained for an antenna distance of 100 mm in the setup of Figure 6, which was the antennas’ closest position to the MM layer considered in these experiments (4 mm). We will refer to this configuration, which is the most significant to the end of integrating the MM inside the headband scanner, as “Distance 1”. These measurements also suggest that, despite the physical limitations of the setup and the presence of the acrylic which results in additional scattering, signal differences due to the MM are still detectable.

**Figure 9:**
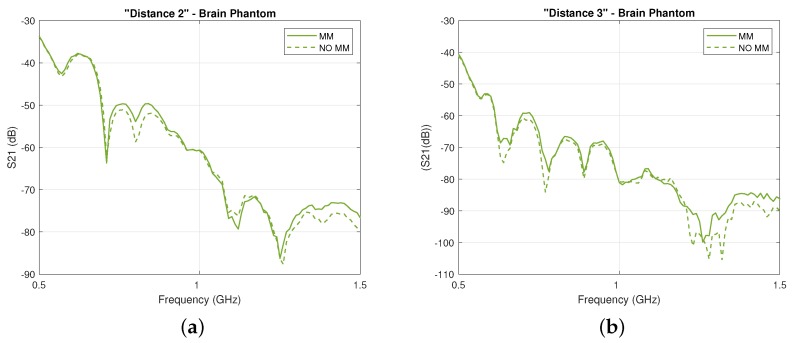
Experimentally measured transmission coefficient $S_{21}$ with the MM (solid lines) and without the MM (dashed lines) when the inner layer is filled with the brain phantom for (**a**) “Distance 2” and (**b**) “Distance 3”.

For the first case scenario (“Layer 3” filled with brain phantom), as shown in Figure 9a,b, we measured transmission for other two different distances between the antennas: 110 mm and 130 mm, corresponding respectively to Antenna 1 placed at 10 mm (“Distance 2”) and at 20 mm (“Distance 3”) from the MM. In particular, we first measured the S21 parameter for all the distances with the MM. Then, we removed it and repeated the measurements. Thus, even if the antenna spacing was changing, the stability of the measurements was not significantly affected by the action of placing and removing the MM in front of Antenna 1.

**Figure 10:**
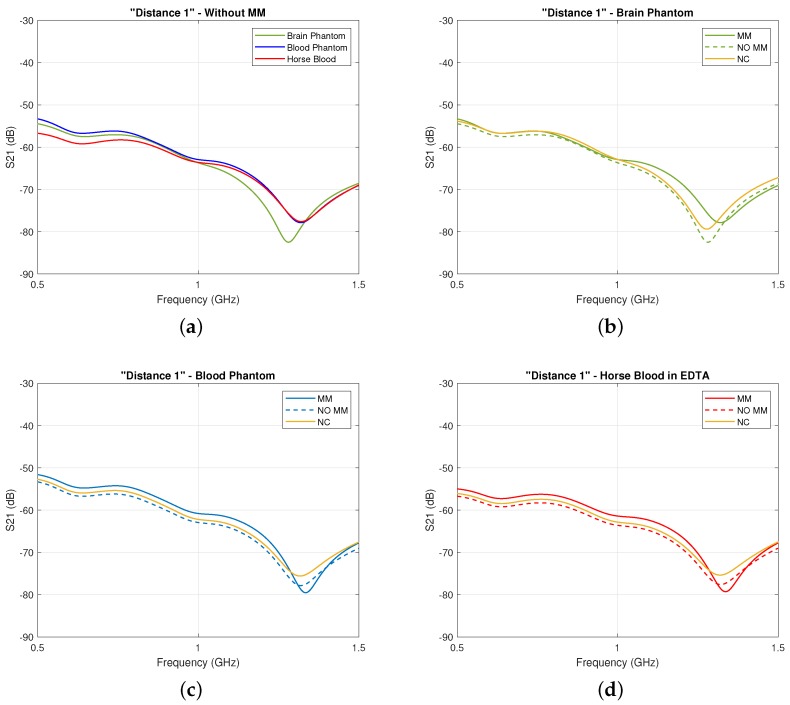
Same plots as Figure 8 for our simulations: (**a**) Comparison of the calculated transmission coefficient $S_{21}$ without the MM and with inner layer filled with brain phantom, blood phantom, and horse blood; (**b**) comparison of the calculated transmission coefficient $S_{21}$ with the MM (solid lines) and without the MM (dashed lines) when the inner layer is filled with the brain phantom, (**c**) with the blood phantom, and (**d**) with the horse blood in EDTA.

We then confirm the first set of our experimental results with our simulations studies presented in Figure 10, which show an overall slight increase in the $S_{21}$ coefficient in the presence of the MM, ranging from 1.0 to 3.0 dB in the considered frequency range. Importantly, this improvement is not observed for the “Negative Control” material in these plots. These results suggest that, rather than generic wave phenomena such as diffraction or signal attenuation due to the partial replacement of the lossy immersion liquid with a lower-loss dielectric material out of which the MM is made, it is the unit cell design and metallic pattern which are responsible for the increased transmitted signals.

**Figure 11:**
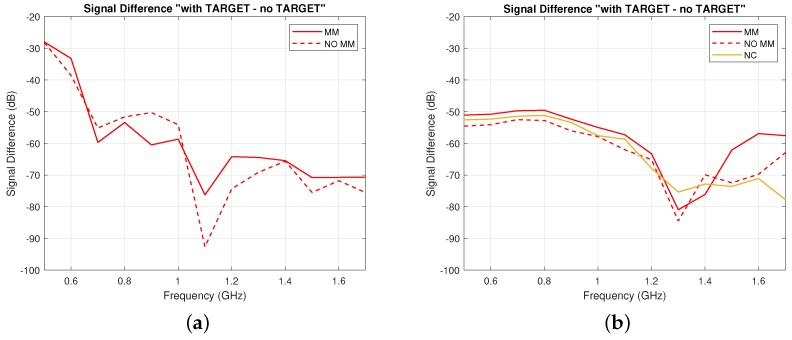
Signal difference “with target – no target” (dB) with (solid lines) and without (dashed lines) the MM, which is due to the blood-filled spherical inclusion in Figure 6: (**a**) experimentally measured and (**b**) calculated from simulations. The same result for the Negative Control (NC) case is plotted in yellow in (**b**).

Finally, our experiments and simulations have also demonstrated that the system is sensitive to changes in permittivity of the inner layer due to the “blood target” inclusion. To illustrate this, the signal difference “with target–no target” was calculated in dB and was shown to be higher in the presence of the MM for both simulations and measurements. As the thickness of the cylindrical shell’s surfaces is small (1 mm) and the ABS’s permittivity is close to the acrylic’s one, the ABS does not significantly affect the detectable signal difference obtained with and without the MM. Experimental results are plotted in Figure 11a and show that the MM increases the signal difference in the 0.5–0.65 GHz and 1.0–1.7 GHz frequency ranges. A very high signal difference of about 16.5 dB was measured around 1.1 GHz. Simulation results in Figure 11a confirm these experimental findings, showing a similar trend and an overall enhancement of the signal difference in the whole frequency range. A NC case was also simulated and led to a lower signal difference than the one obtained with the MM.

### 3.2. Simulation Results with the Headband Scanner

After assessing the MM’s impact with the two-port planar setup, we present results from preliminary simulations of an MWI scanner for h-stroke detection, described in Section 2.3. As in the case of our experiments with the small blood target, we achieved a significant enhancement of the “weak” signal scattered from the target in these simulations. Examples of results are shown below.

**Figure 12:**
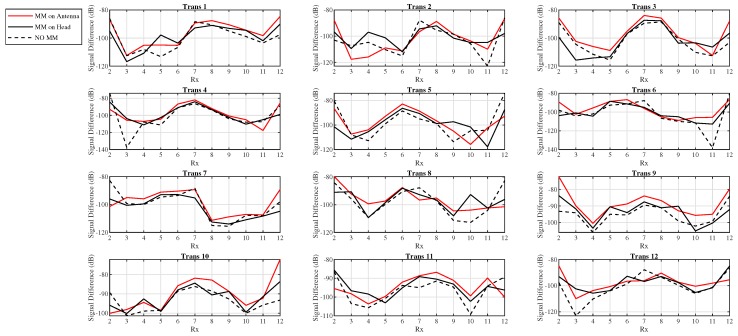
Signal difference “with target–no target” (dB) at 1.5 GHz as a function of receiver location with the MM placed on the antenna’s substrate (red solid lined), with the MM placed on the human head (black solid lines), and without the MM (black dashed lines): As shown in the graph, when the MM is placed on the antenna’s substrate, it leads to higher signal differences with respect to the case in which it is placed on the head.

**Figure 13:**
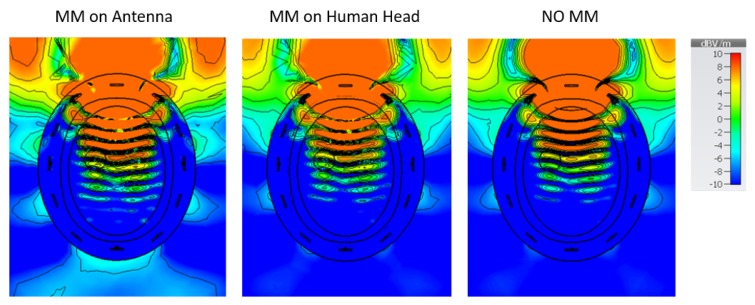
E-fields plotted on a transverse section of the human head model with a blood target in the brain: The MM was placed on the antenna’s substrate (left plot) and on the human head (central plot). On the right is the “no MM case”. A higher field intensity in the target region is shown when the MM is placed adjacent to the antenna’s substrate. In particular, the E-field values calculated inside the target at point P(X,Y) = (29 mm, 29 mm) of the transverse section are 4.8 dB (V/m) and 7.3 dB (V/m) for the MM placed on the head and on the antenna’s substrate, respectively. In the absence of the MM, the intensity of the E-field in the same point is 3.7 dB (V/m).

Figure 12 shows the signal difference “with target – no target” (dB) as a function of receiver location at the antenna’s resonant frequency (1.5 GHz) when the MM is placed both on the head and on the antenna’s substrate. As shown in the graph, when the MM is close to the antenna’s array, it leads to an overall improvement of the signal difference compared to the case in which it is placed on the head (see Transmitters 3, 4, 7, and 9). Furthermore, the E-field plots in Figure 13 show that the MM enhances the intensity of the E-field localized in the target region and that this enhancement is more significant when it is placed adjacent to the antenna’s substrate. As this enhancement translates to higher signal differences due to the target, we further investigated the configuration with the MM film placed on the antenna’s substrate.

**Figure 14:**
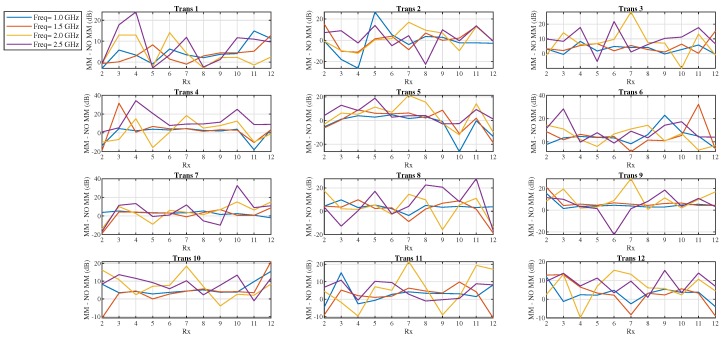
Scattered signal difference “MM–no MM” (dB) as a function of receiver location for the microwave imaging (MWI) scanner of Figure 7, showing an overall improvement of the signal due to the target in the presence of the MM: The signal difference “MM–no MM” is calculated as the difference in dB between the signal difference “with target–no target” (dB) in the presence of the MM and the signal difference “with target–no target” (dB) obtained without the MM.

Figure 14 plots the difference “MM–no MM” (in dB) between the signal difference “with target–no target” (dB) calculated in the presence of the MM on the antenna’s substrate and the signal difference “with target–no target” (dB) obtained without the MM. This difference is plotted as a function of the receiver location for some representative frequencies and is overall positive with several peaks, suggesting that the MM enhances the signal due to the blood target. For instance, the difference “MM–no MM” (dB) for Transmitter 6 is positive for all frequencies and reaches a peak of 30 dB for receivers 11 at the antenna’s resonant frequency of 1.5 GHz.

**Figure 15:**
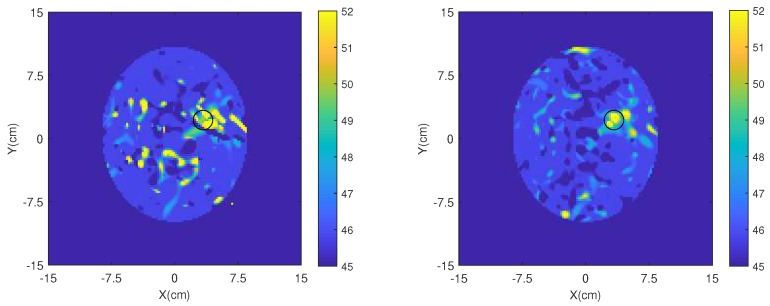
Reconstructed real part of permittivity with frequency hopping without the MM (**left**) and with the MM (**right**).

**Figure 16:**
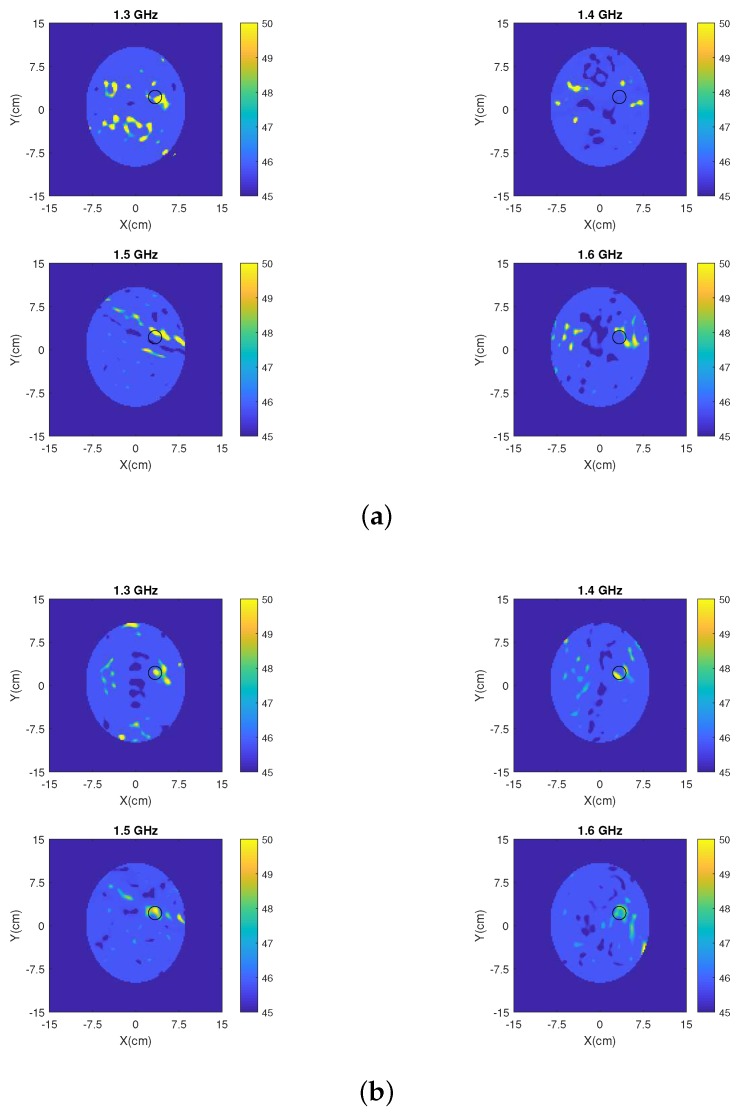
Single frequency reconstruction of the permittivity’s real part for the same data as in Figure 15 at some representative frequencies (1.3 GHz, 1.4 GHz, 1.5 GHz, and 1.6 GHz): (**a**) without the MM and (**b**) with the MM.

Finally, we have applied our 2D DBIM-TwIST algorithm [51,52] to the simulated data plotted in Figure 14 to test the MM’s impact on image reconstructions. To this end, we carried out single-frequency and multi-frequency (frequency hopping) reconstructions, assuming approximate knowledge of the brain tissue and bone as initial guess for our DBIM algorithm. Details of how the algorithm is applied can be found in our previous work [51,52]. Frequency hopping reconstruction results of the estimated dielectric contrast are shown in Figure 15, where five equally spaced frequencies were used in the interval 0.8–2.0 GHz. Comparing the images produced from data with and without the MM shows a clear improvement in image quality when the MM layers are added to each antenna element. In particular, a reduction of artefacts and a better localization of the blood target are observed for the reconstructed permittivity’s real part when the MM is used. The same observation holds true for the single-frequency reconstructions presented in Figure 16.

## 4. Discussion

We have presented preliminary numerical and experimental results which demonstrate the potential of a novel MM structure to enhance MWI systems. The MM unit cell design and two (finite) metasurfaces based on this design were studied in Section 2. To examine the benefits of using the MM as an impedance-matching layer embedded in MWI systems to improve signal transmission though the imaging region, a series of simulation studies were performed to analyse the unit cell’s performance when immersed in a 90% glycerol–water mixture and in contact with the skin. The MM unit cell was shown to improve transmission of about 1.80 dB at 1.0 GHz for plane wave excitation of singular polarization if applied to a simple planar model for the human head.

Subsequently, the two full MM structures were studied. Experimental results showed that a 15 × 18 unit cell MM incorporated in a two-port planar setup improved transmission through a simplified planar head model comprising five different tissue layers. In addition, the MM enhanced the signal scattered from a spherical blood target introduced in the system. Best results were obtained placing Antenna 1 as close as possible to the MM layer, suggesting that the antenna performance was improved by the MM. The presented simulation studies confirmed the experimental findings despite expected differences in the actual transmitted $S_{21}$ coefficients.

Further simulation studies were performed to assess a 5 × 6 unit MM performance incorporated in an MWI system consisting of a 12-antenna array for detecting h-stroke. The MM was placed at various distances between the head skull and the array. Our simulations studies suggest that the MM layer has a better impact when placed adjacent to the substrate of each antenna. In particular, the signal difference due to the blood target inside the brain was shown to increase significantly (up to 30 dB). However, the MM’s impact on the antenna array’s performance needs to be evaluated and quantified to further investigate whether the proposed MM has the potential to act as an antennan MM-based substrate. We then showed that this increased signal difference leads to higher-quality reconstructions of the blood-like target by applying our DIMW-TwIST MWT algorithm. Single and multiple frequency reconstructions showed that the MM can greatly reduce image artefacts and facilitates the localization of the target.

To the best of our knowledge, this is the first paper to show the possibility of improving the efficacy of an MWT system by incorporating an MM structure into its antenna array. These results suggest that metamaterials and metasurfaces can greatly enhance MWT systems, providing a significant hardware advance towards the realisation of MWT systems with the desired clinical accuracy. Our ongoing work is on further experimental studies incorporating the MM in a realistic head scanner with more complex models for the human head, which can provide a more realistic assessment of the benefits from this approach. Furthermore, other clinical applications, such us stroke classification or brain cancer identification and localization, could also benefit from the proposed MM approach and will be investigated in our future research.

## Figures and Tables

**Table 1:** Liquid phantom composition and permittivity at 1 GHz.

Type of Phantom	Water (%)	Glycerol (%)	ϵ’
Skin	30	70	38.2
Bone	10	90	13.2
Brain	35	65	42
Blood	50	50	59.4

## Abbreviations

The following abbreviations are used in this manuscript:

MM	Metamaterial
SRR-CS	Cross-Shaped Split-Ring Resonator
MWT	Microwave Tomography
RF	Radio Frequency
MWI	Microwave Imaging
VNA	Vector Network Analyzer
EDTA	Ethylenediaminetetraacetic Acid
ABS	Acrylonitrile Butadiene Styrene
NC	Negative Control
